# Humanin improves bone health in a glucocorticoid-treated mouse model of Duchenne muscular dystrophy

**DOI:** 10.1016/j.bbrep.2025.102421

**Published:** 2026-01-06

**Authors:** Therése Cedervall, Baptiste Jude, Ferdinand von Walden, Lilly Velentza, Johanna T. Lanner, Thomas Sejersen, Farasat Zaman, Lars Sävendahl

**Affiliations:** a Dept. of Women's and Children's Health, Karolinska Institutet, K6 Kvinnors och barns hälsa, K6 Barnendokrinologi Sävendahl, Stockholm, 171 77, Sweden; b Dept. of Physiology and Pharmacology, Karolinska Institutet, C3 Fysiologi och farmakologi, C3 FyFa Molekylär muskelfysiologi och patofysiologi, Stockholm, 171 77, Sweden; c Center for Neuromusculoskeletal Restorative Medicine, Hong Kong Science Park, Shatin, New Territories, Hong Kong

**Keywords:** Duchenne muscular dystrophy, mdx, Glucocorticoids, Humanin, Growth retardation, Osteoporosis

## Abstract

Duchenne muscular dystrophy (DMD) is a progressive muscle disease for which glucocorticoid (GC) treatment is standard therapy. Patients typically suffer from short stature and osteoporosis, caused by the underlying disease and adverse effects of GCs. We investigated whether the mitochondrial peptide humanin (HNG) could prevent GC-induced growth retardation and osteoporosis in mouse models of DMD.

Male mdx mice (B10.mdx and D2.mdx) were treated with GCs, with/without HNG, from 5 to 9 weeks of age using two different treatment regimens. Tibial growth was monitored by weekly X-ray imaging; growth plates analyzed with immunohistochemistry and histomorphometry; and bone structure examined using peripheral quantitative computed tomography. Effects on skeletal muscle were evaluated by immunohistochemistry, qPCR, and *ex vivo* force measurements.

D2.mdx, but not B10.mdx, showed decreased bone growth and impaired bone structure compared with wild type (WT). D2.mdx also displayed increased growth plate height with lower endogenous humanin expression than D2.WT. GC treatment caused growth retardation and reductions in cortical bone area, thickness, and mineral content. Co-administration with HNG prevented bone growth impairment at one week of treatment and mitigated GC adverse effects on cortical bone in B10.mdx mice. Adding HNG to GCs did not exacerbate skeletal muscle pathology; in fact, HNG had a mild enlarging effect on muscle fibers.

These data suggest that HNG is a potential candidate for improving bone health in DMD during GC therapy. Further *in vivo* studies are needed to determine optimal HNG dosing and to assess the effects of long-term treatment on skeletal muscle function.

## Highlights

• D2.mdx, not B10.mdx, showed compromised bone growth and structure vs. wild type.
• Adding HNG to GC treatment mitigated GC-induced growth suppression the first week.
• HNG mitigated GC's negative effects on cortical bone.
• Adding HNG to GC treatment did not exacerbate muscle pathology.

## 1 Introduction

Duchenne muscular dystrophy (DMD) is an inherited, incurable disease that leads to progressive muscle weakness, loss of ambulation and, eventually, early death due to respiratory or cardiac failure. This disorder is caused by a mutation in the dystrophin gene, which is located on the X chromosome; therefore, it almost exclusively affects males. The incidence is 1 in 3500 to 6000 newborn boys worldwide [1].

In addition to the hardship associated with muscle disease, many patients with DMD also suffer from poor bone health, including both growth retardation resulting in short stature and osteoporosis increasing the risk of fractures of long bones and vertebrae [[2], [3], [4], [5], [6]]. Muscular dystrophy alone negatively affects the skeleton, not only because of reduced mobility but also because of chronic inflammation and hormonal disturbances [7]. The number of untreated DMD patients is overall one standard deviation shorter than that of the average healthy population, and long bone fractures are reported in 21–44 % of patients [[4], [5], [6]]. In addition, long-term treatment with glucocorticoids (GCs), which is currently the standard treatment for slowing the progression of muscle degeneration, adds an average shortage of 20 cm in height, decreases bone mineral density, and increases the risk of vertebral fractures [2,3,8].

Growth failure is a significant concern for most boys with DMD and their families and can negatively impact psychosocial health, quality of life and clinical outcomes [9]. It is a major side effect when daily or intermittent GC regimens are used; intermittent GC treatment has a more favorable side effect profile but is less effective at preserving muscle function [10]. Bone fractures not only are painful but also reduce mobility, and many patients permanently lose their ability to walk after a long-bone fracture [11]. Additionally, vertebral fractures can exacerbate respiratory decline, further decreasing survival [12].

To treat growth failure in boys with DMD, recombinant growth hormone (GH) therapy has been tested in small clinical trials [13,14]. Although GH promotes growth and is well tolerated, reported side effects include insulin resistance and intracranial hypertension [13], which are known adverse effects of GH therapy in other conditions as well [15], [16]. Osteoporosis in DMD is treated with bisphosphonates and dietary supplementation with vitamin D and calcium if daily intake is inadequate [17]. High-quality evidence suggests that bisphosphonates significantly increase the areal lumbar spine bone mineral density (BMD), but strong evidence that bisphosphonates reduce fracture risk in patients with DMD is lacking [18]. Bisphosphonates also have both acute- and long-term adverse effects, including gastrointestinal problems, atypical femur fractures and osteonecrosis of the jaw [19,20]. Another approach is the newly developed GC analog vamorolone, which shows similar efficacy to prednisolone in boys with DMD, without side effects on growth and bone turnover [21]. The results are promising, but long-term data are limited. More options for efficient and safe treatment strategies to improve bone growth and bone quality in DMD patients are therefore highly desired.

Previous studies from our group have shown that treatment with [14 Gly]-humanin (HNG), a potent analog to the mitochondria derived peptide humanin, successfully rescues mice from growth retardation caused by GCs and anticancer drugs [[22], [23], [24]]. Underlying mechanisms of the effects of HNG on chondrocytes include decreased apoptosis and restored proliferation during GC treatment, decreased inflammation, and regulation of the growth plate signaling molecule Indian hedgehog [24]. HNG also regulates the pro-apoptotic protein Bax when it protects bones from chemotherapy-induced toxicity [23]. Humanin has been widely investigated for its neuroprotective and cytoprotective properties and has shown promising therapeutic potential in a variety of tissues and disorders, including Alzheimer's disease, stroke, cardiovascular disease and type 2 diabetes [[25], [26], [27], [28]]. Furthermore, humanin has been linked to energy metabolism and aging [29]. Interestingly, humanin serum levels increase following endurance exercise [30].

In this preclinical study, we investigated the efficacy and safety of HNG in preventing GC-induced growth retardation and osteoporosis in mouse models of DMD. To address this, we administered GCs, either alone or in combination with HNG, to young growing male mdx mice. Although our main objective was to study the effects of HNG on bone health, we also investigated any potential undesired effects in dystrophic muscle.

## 2 Methods

### 2.1 Animals

Two mouse strains, B10.mdx and D2.mdx, were used. B10.mdx is the most established DMD model, whereas the newer D2.mdx carries the same spontaneous mutation in the dystrophin gene but on a different genetic background and displays more pronounced muscle pathology [31]. Breeding pairs of B10.mdx (C57BL/10ScSn-Dmd^mdx^/J), B10. WT (C57BL/10ScSnJ), and D2.mdx (D2. B10-Dmd^mdx^/J) were obtained from Jackson Laboratories. D2.WT (DBA/2J) were obtained from ScanBur and acclimatized for 5 days prior to starting. Only male mice were used in the experiments. The animals were housed under a 12-h light/dark cycle in a temperature- and humidity-controlled environment at the Department of Comparative Medicine at Karolinska Institutet, Stockholm, Sweden. Water and standard chow were provided ad libitum and enrichments for well-fare. A humane endpoint was established and monitored according to the KI's assessment template. All experiments were approved by the local ethical committee (Stockholm North Animal Ethics Committee, permit numbers N110-14 and 13572-2018).

### 2.2 Treatments

Treatments began at 5 weeks of age and continued for 4 weeks for all experiments. Body weight and litter size differences were balanced during group allocation. Consequently, the individual cages contained animals from different treatment groups.

In the B10.mdx mouse experiment, a subcutaneous (s.c.) 2.5 mg slow-release prednisone pellet (60-day continuous release, Innovative Research of America, Sarasota, FL) was inserted under the neck skin at the beginning of the study, using a 10-gauge precision trocar under isoflurane anesthesia [32]. Subcutaneous GC pellets were chosen because this is a well-established model to induce osteoporosis in mdx mice [32,33]. B10.mdx mice that did not receive prednisone were implanted with a placebo pellet instead. The pellets remained implanted until the endpoint. In addition, intraperitoneal (i.p.) injections of 1 mg/kg body weight (BW) synthesized [Gly14]-humanin (HNG; GeneScript, Piscataway, NJ) dissolved in saline (0.9 % NaCl) were given daily. The mice that did not receive HNG received i.p. injections of saline. B10.mdx mice (n = 33) were divided into four groups: control (placebo pellet + saline, n = 9), HNG (placebo pellet + HNG, n = 8), PredP (prednisone pellet + saline, n = 9) and PredP + HNG (prednisone pellet + HNG, n = 7). Wild-type mice B10.WT (n = 10) were not treated and were followed in conjunction with the other groups.

As the growth curves shown in Fig. 3B and C suggest uneven GC release from the pellets, which is consistent with previous reports of unstable serum levels with similar pellets of corticosterone [34,35], we switched to daily i.p. injections of prednisolone for the D2.mdx mouse experiments. This approach allows weight-adjusted dosing, more consistent exposure, with the use of dosages previously shown to reduce tibia length [36].

In the D2.mdx experiment, i.p. injections of 5 or 10 mg/kg BW prednisolone (Sigma Aldrich) dissolved in saline and 100 μg/kg BW HNG were administered daily. D2.mdx mice (n = 61) were divided into six groups: control ( saline; n = 10), HNG (n = 10), Pred5 (5 mg/kg BW prednisolone; n = 11), Pred5+ HNG (5 mg/kg BW prednisolone + HNG; n = 10), Pred10 (10 mg/kg BW prednisolone; n = 10), and Pred10+HNG (10 mg/kg BW prednisolone + HNG; n = 10). Wild-type mice D2.WT (n = 10) were not treated and were followed in conjunction with the other groups.

In an experiment intended solely for *ex vivo* force measurements in skeletal muscle tissue, D2.mdx mice (n = 25) received daily i.p. injections of 1 mg/kg BW prednisolone and/or 100 μg/kg BW HNG. The groups were as follows: control (saline, n = 6), Pred (prednisolone; n = 6), HNG (n = 7) and Pred + HNG (prednisolone + HNG, n = 6). Wild-type mice D2.WT (n = 5) were not treated.

To study the concentration of prednisolone in serum after injection, 10 nine-week-old D2.mdx mice were injected i.p. with 10 mg/kg BW prednisolone. The mice were sacrificed with CO_2_ either 30 min (n = 5) or 24 h (n = 5) after the injection for blood sampling and serum separation.

In total, 154 mice were used across the experiments. Sample sizes were determined based on previous experimental experience using tibial growth (or specific force in the *ex vivo* experiment) as the primary outcome measure. A schematic overview of the two experiments studying bone growth is shown in Fig. 3A and 4A.

### 2.3 Tibial length measurements

X-ray images of the tibia were taken at the start, end and every week with a Mobilett Plus mobile X-ray machine (Siemens Healthineers, Erlangen, Germany; settings: 50 kV, 2.5 mAs), while the animals were lightly anesthetized with 2.5–4 % isoflurane as previously described [24]. Bone length was measured with ImageJ software (National Institutes of Health) in a blinded manner and is the mean length of the left and right tibiae.

### 2.4 Tissue preparation

After termination, the tibias and diaphragm were fixed in 4 % formaldehyde and stored in 70 % EtOH. Gastrocnemius muscle was immediately snap frozen in isopentane on dry ice for storage at −80 °C until analysis. Blood was collected through cardiac puncture in conjunction with euthanasia with CO_2_, and serum was separated by allowing coagulation at RT followed by centrifugation (2 × 10 min at 1500 g).

### 2.5 Growth plate histomorphometry

The bones were decalcified in 10 % EDTA prior to being embedded in paraffin. Sections, 5 μm thick, were cut with a microtome, with each microscope slide containing tissue from all groups to ensure uniform treatment. After deparaffinization and rehydration in a gradient ethanol series from xylene to distilled water, the sections for histomorphometric analysis were stained with Alcian blue solution followed by nuclear fast red solution (Sigma Aldrich). Measurements were performed in a blinded manner using ImageJ software.

### 2.6 Immunohistochemistry and immunofluorescence staining of the growth plate and diaphragm

Immunohistochemistry (IHC) was performed as previously described [37], with anti-humanin (1:200; #NB100-56877; Novus Biol) and anti-Bax (1:100; #SC6236; Santa Cruz) primary antibodies and goat anti-rabbit (1:300; 1:250; #ab97049; Abcam) secondary antibody. Proliferating cell nuclear antigen (PCNA) expression was analyzed by immunofluorescence staining as previously described [38] with a primary rabbit antibody against PCNA (1:1000; #ab18197; Abcam) and a FITC-conjugated secondary antibody (1:250; #F2765; Thermo Fisher). Quantification was performed with ImageJ or manual counting of cells in a blinded manner.

### 2.7 Peripheral quantitative computed tomography (pQCT)

The bone geometry and mineral content in the dissected tibias were analyzed with peripheral quantitative CT XCT RESEARCH M (version 4.5B; Norland) operating at a resolution of 70 μm, as described previously [39]. Cortical bone parameters were analyzed in the mid diaphyseal region, whereas trabecular bone parameters were analyzed in the proximal metaphyseal region of the same bone.

### 2.8 Skeletal muscle histology

Transverse sections of the gastrocnemius (10 μm) were incubated with wheat germ agglutinin (WGA) conjugated with Texas Red (TX-Red) (#W21405; Invitrogen), and the nuclei were stained with mounting media containing DAPI (#00-4959-52; Invitrogen). Slices were imaged with the Axioscan.Z1 (ZEISS), cross-sectional area (CSA) and the percentage of muscle fibers with central nuclei were analyzed with MuscleJ, a new ImageJ/Fiji tool [40]. The analysis was performed on whole muscle sections (2000–4000 fiber per muscle section).

### 2.9 RT‒qPCR

Total RNA was isolated from 15 mg of gastrocnemius muscles with TRI Reagent (Sigma‒Aldrich) according to the manufacturer's recommendations. After TURBO DNAse (Invitrogen) treatment, cDNA was synthesized from 500 ng of RNA using dNTP mix, Oligo(dT)_20_ primer and a Superscript IV reverse transcriptase kit (Invitrogen). qPCR was performed on cDNA diluted 1/20 (1/100 for 18S), primers at a final concentration of 0.4 μM, with the iTaq Universal SYBR Green Supermix (Bio-Rad). Gene expression was analyzed with the ΔΔCt method, and 18S was used as a housekeeping gene. The primers used are detailed in Table 1.

**Table 1:** List of primers used in RT-qPCR.

Gene	Forward	Reverse
18S	AGTCCCTGCCCTTTGTACACA	CGATCCGAGGGCCTCACT
TNF-α	CAGGCGGTGCCTATGTCTC	CGATCACCCCGAAGTTCAGTAG
IL-6	ACAAAGCCAGAGTCCTTCAGAG	AGAGCATTGGAAATTGGGGTAGG
PGC-1α1	GGACATGTGCAGCCAAGACTCT	CACTTCAATCCACCCAGAAAGCT
ERRα	ACCCTTTGCCTTTCCCG	CGTACAGCTTCTCAGGTTCAAC
Cs	TGGGAGCCAAGAACTCATCCT	CCATGTTGCTGCTTGAAGGTC
Tfam	CACCCAGATGCAAAACTTTCAG	CTGCTCTTTATACTTGCTCACAG
Col1a1	CGATGGATTCCCGTTCGAGT	CGATCTCGTTGGATCCCTGG
Mstn	AGTGGATCTAAATGAGGGCAGT	GTTTCCAGGCGCAGCTTAC

### 2.10 *Ex vivo* force measurements

*Ex vivo* force measurements were performed on intact extensor digitorum longus (EDL) and soleus (Sol) muscle as previously described [41] and on a section of the diaphragm using the “suture approach” [42]. Muscles were dissected and then transferred to the force measurement system and incubated in Tyrode solution (121 mM NaCl, 5 mM KCl, 1.8 mM CaCl_2_, 0.4 mM NaH_2_PO_4_, 0.5 mM MgCl_2_, 24 mM NaHCO_3_, 0.1 mM EDTA, and 5.5 mM glucose) at 31 °C and gassed with 95 % O_2_ and 5 % CO_2_. Muscles were set to the optimal L_0_ stimulated with a supramaximal pulse (1 ms at 15 V). Following a 15 min equilibration period in the Tyrode solution, they were stimulated at different frequencies every minute: 1–120 Hz, 1 s duration for the soleus, and 1–150 Hz, 300 ms duration for the EDL and diaphragm bundles. The quantified force was expressed as the specific force, which was calculated by dividing the absolute force recorded by the cross-sectional area (muscle mass divided by muscle length and density (1.06 g/cm^3^).

### 2.11 Creatine kinase (CK) activity

Serum CK activity was measured using a commercial Creatine Kinase Activity Colorimetric Assay Kit (#K777-100 BioVision) according to the manufacturer's instructions.

### 2.12 Mass spectrometry

The concentration of prednisolone in serum samples was measured by mass spectrometry. For more details, refer to supplementary text S1.

### 2.13 Statistical analysis

All the statistical analyses were performed in GraphPad Prism (GraphPad Software LLC). Comparisons between two groups (WT vs. mdx) were analyzed with Student's unpaired *t*-test. Comparisons between several groups (treatments) were analyzed with one-way ANOVA and Tukey's multiple comparison test. When repeated measures were performed for the same subject and two or more groups were compared, mixed-model ANOVA was used. Required assumptions of normal distribution and homogeneity of variance were analyzed with the software. The CK analysis data were not normally distributed; therefore, nonparametric Mann‒Whitney (WT vs. mdx) and Kruskal‒Wallis tests (between treatments) were applied. A p value < 0.05 was considered to indicate statistical significance. No specific exclusion criteria were applied to the data, and all the obtained data points were included in the analysis.

## 3 Results

### 3.1 Natural bone phenotype in mdx and wild-type mice

#### 3.1.1 Tibial growth

**Fig. 1:**
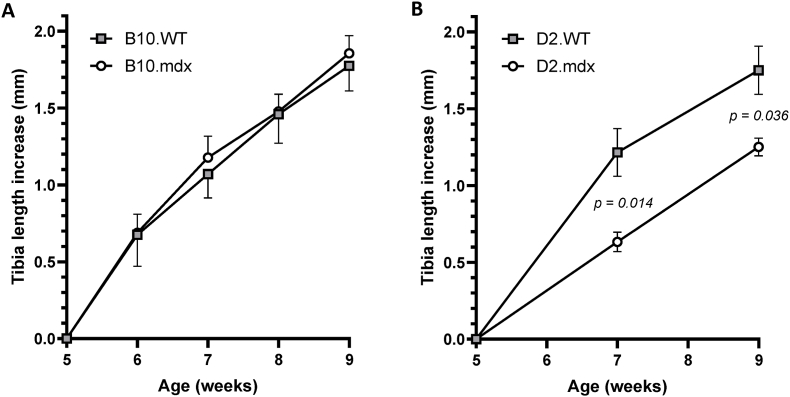
The increase in tibia length between 5 and 9 weeks of age in mdx and wild-type mice on the two different genetic backgrounds, B10 **(A)** and D2 **(B)**. Mean values ± SEM, n = 8–10, mixed-model ANOVA.

In B10.mdx mice, tibial bone growth between 5 and 9 weeks of age was similar to that in B10.WT mice (Fig. 1A). During this period, the length of the tibia increased with 1.86 ± 0.12 mm in B10.mdx and 1.78 ± 0.16 mm in B10.WT. In contrast, tibial bone growth was suppressed in D2.mdx mice compared with D2.WT mice. The tibia length of D2.mdx mice increased by only 1.25 ± 0.06 mm, whereas the length increased by 1.75 ± 0.16 mm in D2.WT mice (Fig. 1B).

#### 3.1.2 Body weight

Although there was no difference in tibia growth among the B10 strains, the body weight increased by 10.6 ± 0.57 g in B10.mdx but increased by only 6.1 ± 0.66 g in B10.WT (p = 0.0004). In D2.mdx mice, the suppressed tibia bone growth was accompanied by a lower body weight gain compared with D2.WT mice (3.5 ± 0.5 g versus 7.6 ± 0.6 g, respectively (p = 0.0001)).

#### 3.1.3 Growth plate morphology and humanin expression

**Fig. 2:**
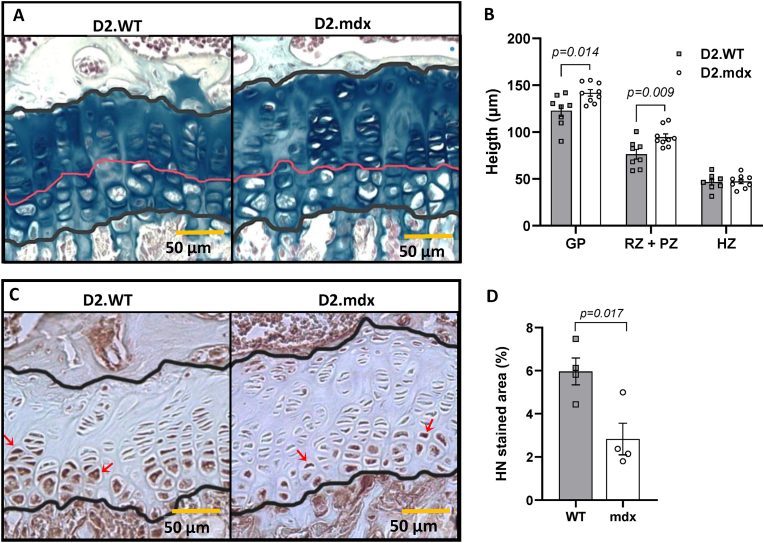
Pictures of growth plate tissue from 9-week-old D2.WT and D2.mdx mice **(A)** and measurements of the total growth plate (GP), resting zone (RZ) + proliferative zone (PZ) and hypertrophic zone (HZ) height **(B)**. Pictures and quantification of endogenous humanin (HN) expression in growth plates stained with IHC and DAB **(C, D)**. Red arrows point at stained chondrocytes. Mean values ± SEM, histology: n = 8–10 mice; humanin expression: n = 4 mice, Student's unpaired *t*-test.

The D2.mdx mice displayed morphological changes in their growth plates. The height of the growth plate was greater in D2.mdx mice, which was attributed to a greater height of the resting plus proliferative zone (Fig. 2A and B). The number of chondrocyte columns was similar, with 26.6 ± 2.3 columns/mm for WT and 31.9 ± 2.8 for mdx. The expression of the proliferative marker PCNA did not differ significantly, with 10.6 ± 1.9 % of cells positive for PCNA expression in D2.WT mice and 6.9 ± 1.3 in D2.mdx mice. In addition, no significant difference in the percentage of pro-apoptotic protein Bax-positive cells in the hypertrophic zone was noted with values of 50.3 ± 11.0 % positive cells in D2.WT mice and 48.7 ± 4.3 in D2.mdx mice. D2.mdx mice displayed significantly lower protein levels of endogenous humanin in the growth plate (Fig. 2C and D). Humanin staining was observed mainly in the hypertrophic zone in both mdx- and WT mice.

**Fig. 3:**
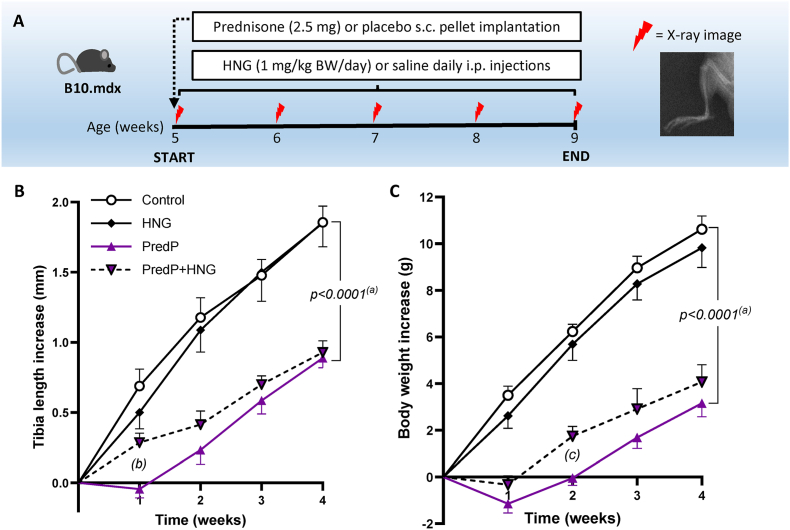
Schematic view **(A)** of the experiment in B10.mdx mice treated with either 2.5 mg prednisone s.c. pellet (PredP) and/or i.p. injections with 1 mg/kg BW/day HNG. The control group is B10.mdx mice receiving placebo pellet and saline. Weekly X-rays were taken to measure the longitudinal bone growth and the increase in tibia length is presented in **(B)** and body weight in **(C).** Mean ± SEM, n = 7–9, mixed-model ANOVA. P-values refer to: (a) PredP vs. control; (b) PredP + HNG vs. PredP p = 0.014; (c) PredP + HNG vs. PredP p = 0.023.

**Fig. 4:**
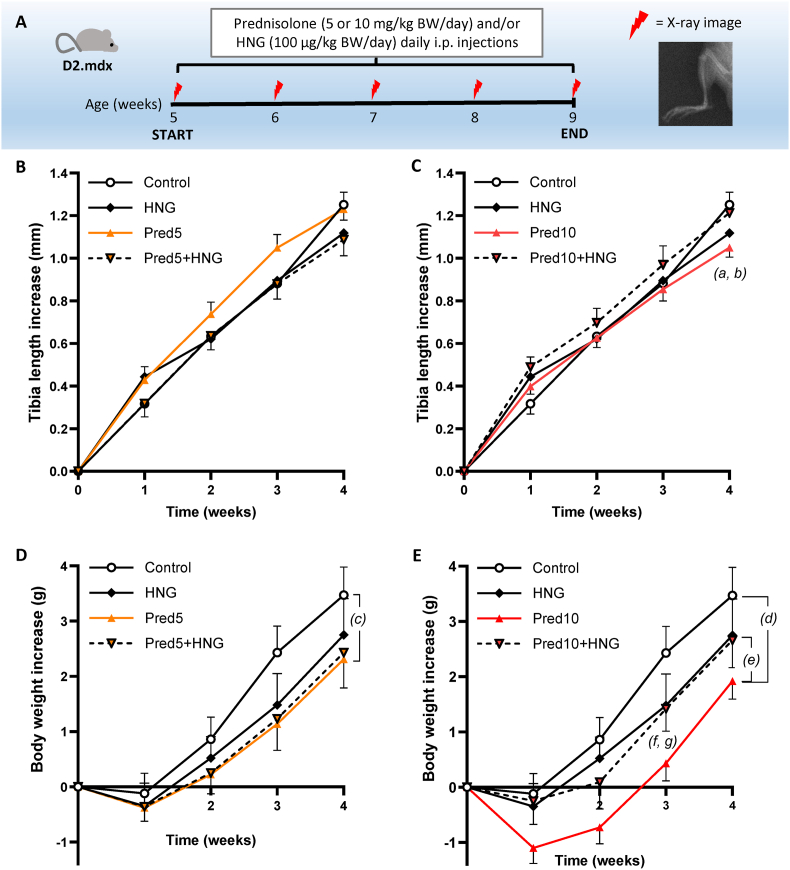
Schematic view of the experiment in D2.mdx mice administered 5 or 10 mg/kg BW/day prednisolone (Pred5, Pred10) and/or 100 μg/kg BW/day HNG with i.p. injections (controls = D2.mdx injected with saline) **(A)**. Weekly X-rays were taken to measure the longitudinal bone growth. Tibia length increase **(B, C)** and body weight **(D, E)**. The two doses of prednisolone are separated into different graphs for better visualization. Mean values ± SEM, n = 9–11, mixed-model ANOVA. P-values refers to: (a) Pred10 vs. control p = 0.12; (b) Pred10+HNG vs. Pred10 p = 0.49; (c) Pred5 vs. control p = 0.61; (d) Pred10 vs. control p = 0.16; (e) Pred10+HNG vs. Pred10 p = 0.81; (f) Pred10 vs. control p = 0.031; (g) Pred10+HNG vs. Pred10 p = 0.42.

#### 3.1.4 Bone geometry and mineral density

The cortical thickness, area and mineral content was reduced in the D2.mdx tibias compared to D2.WT along with the periosteal and endosteal circumferences (Table 2). A reduction in total BMD was noted. Similar differences were not observed in the B10.mdx mouse strain.

**Table 2:** Bone parameters in two different strains of mdx and wild-type mice.

	B10.WT	B10.mdx	D2.WT	D2.mdx
	n = 10	n = 9	n = 10	n = 10
Length (mm)	17.21 ± 0.07	17.01 ± 0.07 ^*p= 0.056 (a)*^	16.96 ± 0.12	16.83 ± 0.11
Tot.BMD (mg/cm^3^)	337 ± 90	346 ± 11	398 ± 90	378 ± 12 ^*p = 0.0003 (b)*^
Tr.BMD (mg/cm^3^)	216 ± 13	233 ± 16	317 ± 18	270 ± 18 ^*p = 0.071 (b)*^
Crt.BMD (mg/cm^3^)	1004 ± 10	991 ± 13	1093 ± 90	1109 ± 70
Crt. Area (mm^3^)	0.656 ± 0.026	0.651 ± 0.032	0.592 ± 0.023	0.493 ± 0.011 ^*p = 0.001 (b)*^
Crt. Thickness (mm)	0.153 ± 0.006	0.155 ± 0.006	0.201 ± 0.007	0.181 ± 0.003 ^*p = 0.017 (b)*^
Crt. CNT (mg/mm)	0.660 ± 0.031	0.648 ± 0.037	0.653 ± 0.029	0.550 ± 0.015 ^*p = 0.006 (b)*^
Peri.C (mm)	4.77 ± 0.04	4.67 ± 0.07	3.59 ± 0.04	3.30 ± 0.05 ^*p = 0.0003 (b)*^
Endo.C (mm)	3.81 ± 0.05	3.70 ± 0.04 ^*p = 0.09 (a)*^	2.33 ± 0.02	2.16 ± 0.05 ^*p = 0.006 (b)*^
PMI (mm^4^)	0.273 ± 0.015	0.259 ± 0.023	0.110 ± 0.007	0.077 ± 0.004 ^*p = 0.0009 (b)*^
MR (mm^3^)	0.234 ± 0.014	0.233 ± 0.017	0.150 ± 0.009	0.119 ± 0.006 ^*p = 0.011 (b)*^

pQCT analysis of tibiae bones from 9-week-old mdx and wild-type mice on two genetic backgrounds: volumetric total bone mineral density (Tot.BMD); volumetric trabecular BMD (Tr.BMD); volumetric cortical BMD (Crt.BMD); cortical area, thickness and mineral content (Crt.CNT); periosteal circumference (Peri.C); endocortical circumference (Endo.C); calculated polar moment of inertia (PMI) and moment of resistance (MR). Mean values ± SEM, Student's unpaired *t*-test, (a) p-value vs. B10.WT; (b) p-value vs. D2.WT.

### 3.2 Bone phenotype in mdx mice treated with GCs

#### 3.2.1 Bone growth and body weight

The implantation of a subcutaneous prednisone pellet had a negative effect on both tibia growth and body weight in B10.mdx mice (Fig. 3). Decreased growth was observed after 1 week and was maintained throughout the treatment.

In D2.mdx mice, neither 5 mg prednisolone/kg BW/day (Pred5) nor 10 mg prednisolone/kg BW/day (Pred10), caused growth retardation in the tibiae (Fig. 4B and C). The Pred10 group had significantly decreased body weight gain at 3 weeks of treatment, whereas no difference in weight gain was detected for Pred5 (Fig. 4D and E). No differences in growth plate morphology or proliferation (PCNA) or apoptosis (Bax) markers were detected when the Pred10 group was analyzed (Table 3).

**Table 3:** Growth plate histomorphometry and IHC in prednisolone- and HNG-treated D2.mdx mice.

	Control	HNG	Pred10	Pred10 + HNG
Histomorphometry				
Total growth plate height (μm)	141.9 ± 3.5	141.6 ± 7.3	141.9 ± 4.6	128.7 ± 5.0
Resting + Proliferative zone height (μm)	94.6 ± 3.5	97.8 ± 6.5	99.8 ± 4.0	88.1 ± 3.8
Hypertrophic zone height (μm)	47.3 ± 2.4	43.8 ± 2.5	42.1 ± 1.5	40.6 ± 1.8
Number of columns (n/mm)	31.9 ± 2.8	33.5 ± 1.2	27.1 ± 2.4	30.9 ± 1.8
IHC				
PCNA-positive cells (%)	6.9 ± 1.3	9.3 ± 1.8	8.0 ± 0.9	8.5 ± 1.7
Bax-positive hypertrophic cells (%)	48.7 ± 4.3	45.4 ± 6.5	45.4 ± 5.6	30.0 ± 3.3

The total height of the growth plate, the height of the different zones and the number of cell columns per mm growth plate width in treated D2.mdx (10 mg/kg BW/day i.p. injections of prednisolone and/or 100 μg/kg BW/day HNG, control = D2.mdx receiving saline). Chondrocytes were considered hypertrophic if they had a height ≥7 μm. A cell column was defined as a columnar structure of ≥5 chondrocytes. Mean values ± SEM, n = 9–10 for histomorphometry, n = 6–10 for IHC, one-way ANOVA (no significant differences).

#### 3.2.2 Bone geometry and mineral density

**Fig. 5:**
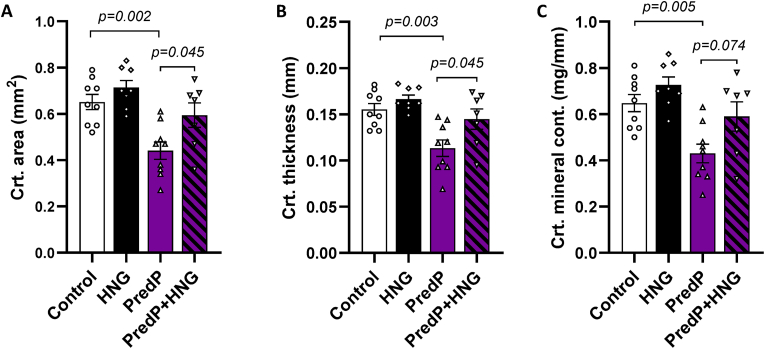
Cortical area **(A)**, thickness **(B)** and mineral content **(C)** in B10.mdx mice treated with either a 2.5 mg prednisone pellet (PredP) and/or 1 mg/kg BW/day HNG (control group = B10.mdx treated with placebo pellet and saline). Mean values ± SEM, n = 7–9, one-way ANOVA.

Several parameters for bone geometry and mineral density were negatively affected by GCs. The prednisone pellet decreased the cortical area and thickness as well as the cortical mineral content in B10.mdx mice (Fig. 5, full pQCT analysis Table 4). In D2.mdx mice, both the Pred5 and Pred10 groups had decreased cortical area, thickness, and mineral content and decreased total and cortical bone mineral density (Table 5).

**Table 4:** Bone parameters in prednisone- and HNG-treated B10.mdx mice.

	Control	HNG	PredP	PredP + HNG
	n = 9	n = 8	n = 9	n = 7
Length (mm)	17.01 ± 0.07	17.21 ± 0.10	16.31 ± 0.16 ^*p = 0.002 (a)*^	16.64 ± 0.16
Tot.BMD (mg/cm^3^)	346 ± 11	351 ± 90	329 ± 10	354 ± 19
Tr.BMD (mg/cm^3^)	233 ± 16	247 ± 17	224 ± 13	249 ± 18
Crt.BMD (mg/cm^3^)	991 ± 13	1014 ± 80	968 ± 11	979 ± 22
Peri.C (mm)	4.67 ± 0.07	4.80 ± 0.10	4.24 ± 0.07 ^*p = 0.003 (a)*^	4.54 ± 0.10 ^*p = 0.076 (b)*^
Endo.C (mm)	3.70 ± 0.044	3.75 ± 0.07	3.53 ± 0.06	3.63 ± 0.06
PMI (mm^4^)	0.259 ± 0.023	0.299 ± 0.027	0.137 ± 0.017 ^*p = 0.004 (a)*^	0.223 ± 0.030 ^*p = 0.080 (b)*^
MR (mm^3^)	0.233 ± 0.017	0.258 ± 0.014	0.140 ± 0.016 ^*p = 0.004 (a)*^	0.213 ± 0.026 ^*p = 0.044 (b)*^

Bone morphology measured with pQCT (parameter abbreviations explained in Table 1) in B10.mdx mice treated with a 2.5 mg s.c. prednisone pellet (PredP), with or without HNG (1 mg/kg BW/day). Mean values ± SEM, n = 7–9, one-way ANOVA, (a) p-value vs. control; (b) p-value vs. PredP.

**Table 5:** Bone parameters in prednisolone- and HNG treated D2.mdx mice.

	Control	HNG	Pred5	Pred5+HNG	Pred10	Pred10+HNG
	n = 10	n = 10	n = 11	n = 10	n = 10	n = 10
Length (mm)	16.83 ± 0.11	16.67 ± 0.13	16.58 ± 0.15	16.50 ± 0.12	16.36 ± 0.13	16.36 ± 0.11
Tot. BMD (mg/cm^3^)	378.1 ± 11.8	334.1 ± 8.2 ^*p = 0.054*^	331.5 ± 10.1 ^*p = 0.027*^	352.8 ± 12.2	311.8 ± 9.5 ^*p = 0.0006*^	300.8 ± 10.4
Tr. BMD (mg/cm^3^)	269.5 ± 17.6	235.6 ± 16.4	245.1 ± 17.0	267.9 ± 16.1	212.7 ± 15.3	208.8 ± 19.2
Crt. BMD (mg/cm^3^)	1109.0 ± 7.1	1084.0 ± 6.5	1043.0 ± 15.1 ^*p = 0.002*^	1053.0 ± 17.9	1052.0 ± 7.7 ^*p = 0.013*^	1020.0 ± 8.2
Crt. Area (mm^2^)	0.493 ± 0.011	0.469 ± 0.011	0.414 ± 0.009 ^*p = 0.0003*^	0.451 ± 0.014	0.389 ± 0.014 ^*p < 0.0001*^	0.391 ± 0.014
Crt. Thickness (mm)	0.181 ± 0.003	0.172 ± 0.003	0.149 ± 0.004 ^*p < 0.0001*^	0.161 ± 0.005	0.144 ± 0.004 ^*p < 0.0001*^	0.143 ± 0.004
Crt. CNT (mg/mm)	0.550 ± 0.015	0.508 ± 0.014	0.430 ± 0.015 ^*p < 0.0001*^	0.476 ± 0.022	0.408 ± 0.015 ^*p < 0.0001*^	0.398 ± 0.015
Peri.C (mm)	3.299 ± 0.052	3.264 ± 0.047	3.253 ± 0.023	3.296 ± 0.033	3.140 ± 0.030 ^*p = 0.061*^	3.163 ± 0.043
Endo.C (mm)	2.161 ± 0.053	2.181 ± 0.047	2.321 ± 0.032 ^*p = 0.049*^	2.281 ± 0.035	2.234 ± 0.021	2.263 ± 0.039
PMI (mm^4^)	0.077 ± 0.004	0.072 ± 0.004	0.076 ± 0.006	0.083 ± 0.003	0.056 ± 0.003 ^*p = 0.004*^	0.054 ± 0.003
MR (mm^3^)	0.119 ± 0.006	0.107 ± 0.004	0.081 ± 0.008 ^*p = 0.001*^	0.093 ± 0.010	0.089 ± 0.004 ^*p = 0.022*^	0.083 ± 0.004

Bone morphology measured with pQCT in treated D2.mdx (5 or 10 mg/kg BW/day i.p. injections of prednisolone and/or 100 μg/kg BW/day HNG, control = D2.mdx receiving saline). Mean values ± SEM, n = 10–11, one-way ANOVA, p-values presented are vs. control group. No significant differences were found between Pred5 vs. Pred5+HNG and Pred10 vs. Pred10+HNG.

### 3.3 Effects of HNG on GC-induced bone growth retardation

#### 3.3.1 Bone growth and body weight

When B10.mdx mice were cotreated with PredP and HNG (1 mg/kg BW/day), bone growth significantly improved after one week of treatment but not in the following weeks (PredP + HNG vs. PredP; Fig. 3B). The reduction in body weight gain observed in PredP-treated B10.mdx mice was also partly restored at 2 weeks in the cotreatment group (PredP + HNG vs. PredP; Fig. 3C). In D2.mdx mice, cotreatment with prednisolone and HNG (100 μg/kg BW/day) did not significantly affect body weight gain or tibia bone elongation (Fig. 4B–E) compared with prednisolone alone.

#### 3.3.2 Bone geometry and mineral density

The negative effects of prednisone on cortical thickness and cortical area were counteracted by HNG in B10.mdx mice (Fig. 5). On the other hand, in D2.mdx mice, cotreatment with HNG did not prevent prednisolone-induced side effects on cortical bone (Table 5).

### 3.4 Effects on skeletal muscle

#### 3.4.1 Muscle integrity

**Fig. 6:**
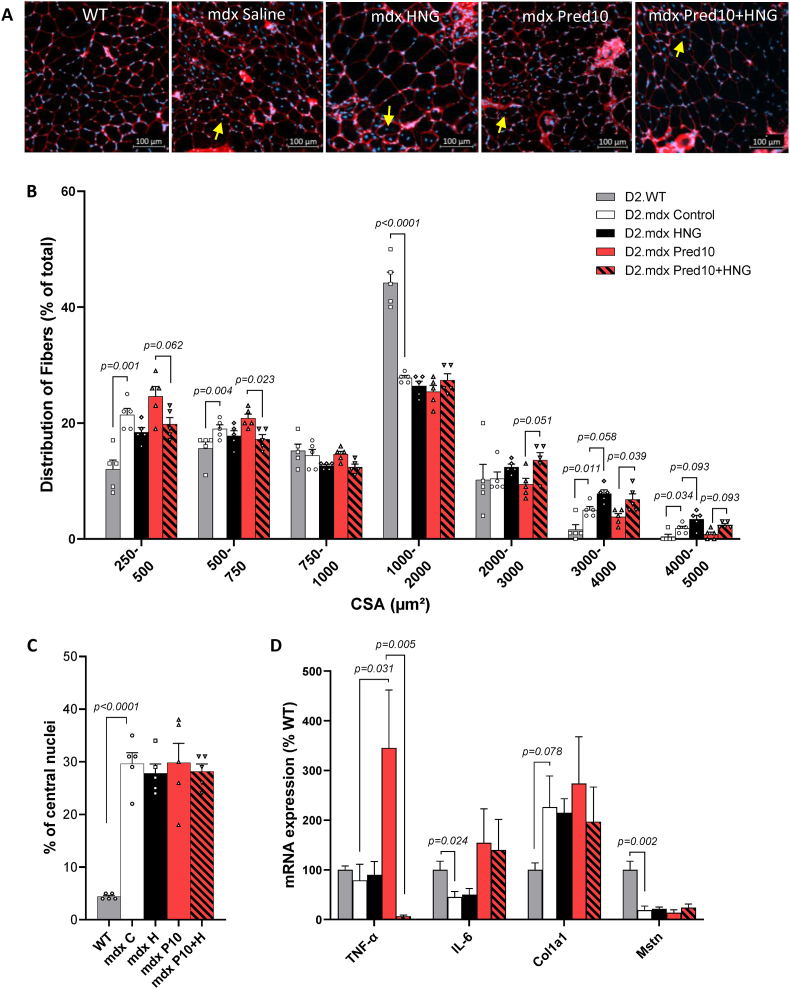
Histological assessment and gene expression analysis in gastrocnemius muscle tissue from D2.WT and treated D2.mdx mice (10 mg/kg BW/day i.p. injections of prednisolone and/or 100 μg/kg BW/day HNG, control = saline). Pictures of WGA/TX-Red stained sections, yellow arrows indicate central nuclei (**A**). The fiber size distribution **(B)** and percentage of cells with central nuclei (**C**). mRNA-expression of inflammatory cytokines TNF-α, IL-6, type 1 collagen COL1A1 and the myokine myostatin, Mstn **(D)**. Mean ± SEM, panel B and C: n = 5; panel D: n = 6, Student's unpaired *t*-test for D2.WT vs. D2.mdx control, one-way ANOVA for comparison between mdx-groups.

D2.mdx mice exhibited a significantly altered fiber size distribution pattern compared with D2.WT mice (Fig. 6A and B). Most of the fibers in D2.WT mice were in the medium size range, between 1000 and 2000 μm^2^, representing 44 % of all fibers. In contrast, only 26 % of the medium-sized fibers were found in D2.mdx mice. In D2.mdx mice, the proportion of small fibers (250–500 μm^2^) was nearly double that in D2.WT mice. This feature was exacerbated in the Pred10 group compared with the D2.WT group. Pred10+HNG treatment resulted in lower proportion of small fibers observed with Pred10 alone, reaching statistical significance in the 500–750 μm^2^ range and borderline significance in the 250–500 μm^2^ range. A greater proportion of large fibers (3000–4000 and 4000–5000 μm^2^) were noted in D2.mdx mice compared with D2.WT mice. Compared with Pred10, the Pred10+HNG group displayed a greater proportion of fibers in the 3000–4000 μm^2^ range, and HNG alone tended to result in greater proportions of large fibers. The number of fibers with central nuclei was approximately sixfold greater in the D2.mdx muscle compared to D2.WT muscle (Fig. 6C). Neither Pred10 nor HNG significantly influenced central nucleation.

#### 3.4.2 Creatine kinase

**Fig. S1:**
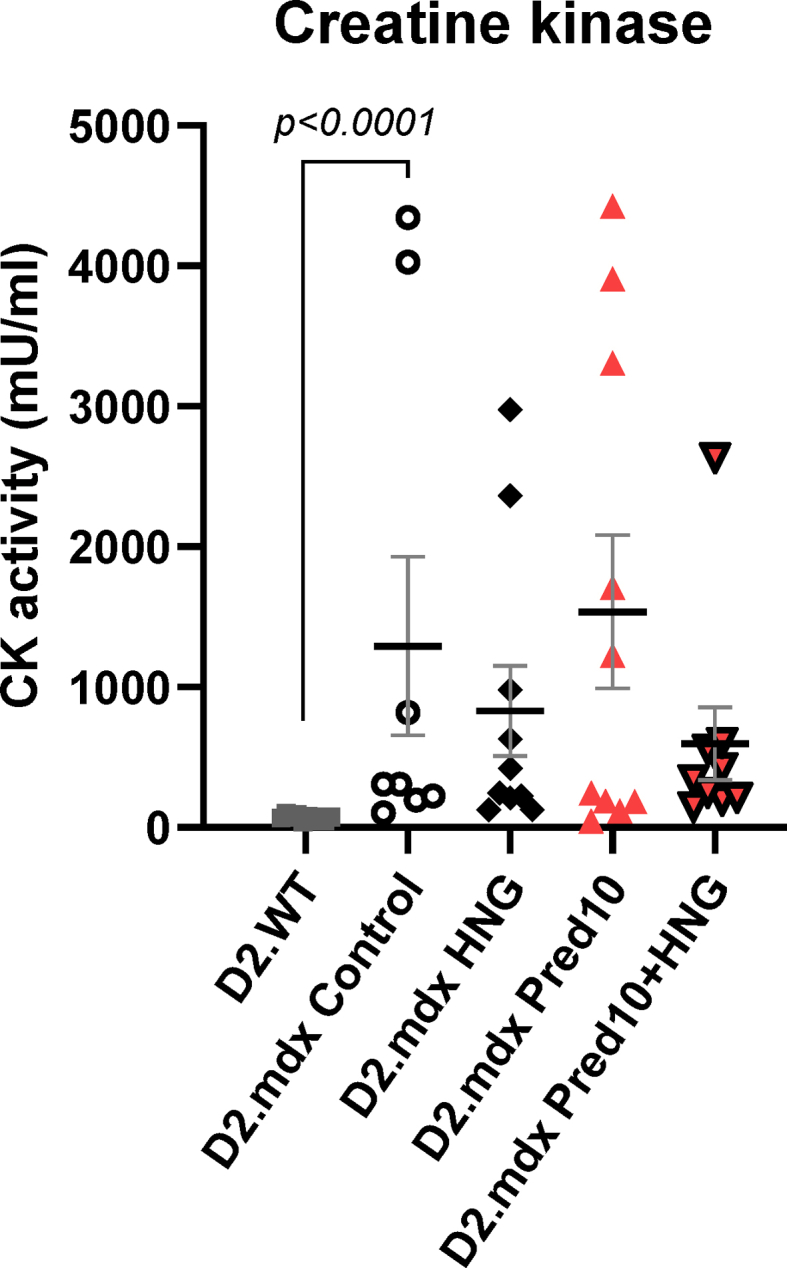
Creatine kinase activity in serum from blood samples collected at endpoint from D2.WT and treated D2.mdx mice (10 mg/kg BW/day i.p. injections of prednisolone and/or 100 μg/kg BW/day HNG, control = saline). Individual values with mean ± SEM, n = 8-10, Mann-Whitney nonparametric test D2.WT vs. D2.mdx control.

Creatine kinase (CK) is crucial for energy metabolism and functions as a marker for muscular dystrophy given that damaged muscle tissue leaks CK into the serum. Analysis of serum samples collected at the endpoint revealed increased CK activity in mdx mice but no effects of the different treatments (Fig. S1).

#### 3.4.3 Gene expression and endogenous humanin

**Fig. S2:**
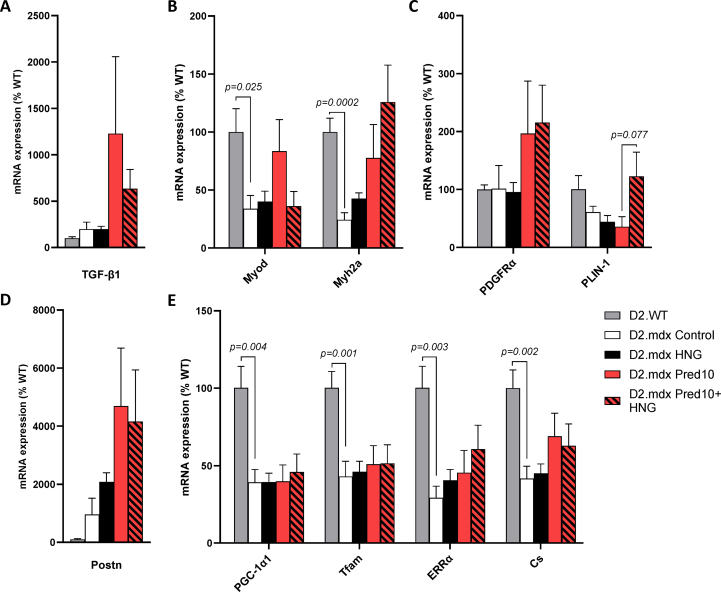
Gene expression in gastrocnemius from D2.WT and treated D2.mdx mice (10 mg/kg BW/day i.p. injections of prednisolone and/or 100 μg/kg BW/day HNG, control = saline). Marker(s) of inflammation: TGF-β1 (A); muscle repair and muscle function: Myod and Myh2a (B); fibrosis and fat infiltration: PDGFRα, PLIN-1 and Postn (C, D); mitochondrial function and oxidative metabolism: PGC-1α1, Tfam, ERRα, and Cs (E). Mean ± SEM, n = 6.

**Fig. S3:**
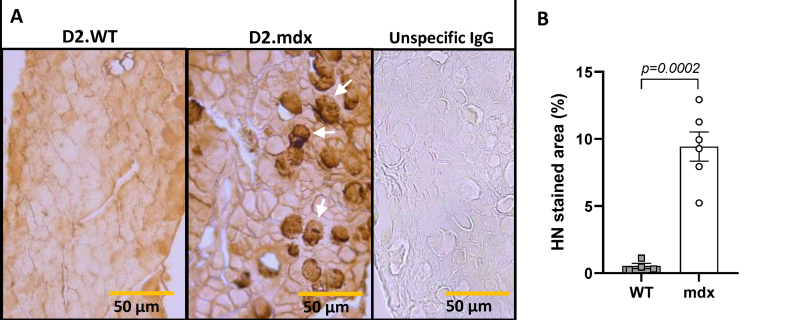
Pictures (A) and quantification (B) of endogenous humanin expression in D2.WT and D2.mdx mice diaphragm, stained with IHC and DAB. White arrows indicate intensely stained areas considered positive. Mean values ± SEM, n = 4-6, Student’s unpaired t-test.

Skeletal muscle gene expression was affected mainly by genotype but also by treatment (Fig. 6D and S2). Specifically, the mRNA expression of the inflammatory cytokine TNF-α was ∼3.5-fold greater in the Pred10-treated group than in the control group, but this expression normalized in the Pred10+HNG group (Fig. 6D). TNF-α levels were similar between D2.WT and D2.mdx control mice, and IL-6 levels were lower in the D2.mdx control mice compared with D2.WT. Key mediators of skeletal muscle homeostasis, such as myostatin (Mstn (Fig. 6D)), markers of muscle function and repair (Myod and Myh2a (Fig. S2B)) and markers of mitochondrial biogenesis and function (PGC-1α1, Tfam, ERRα, and CS (Fig. S2E)), were downregulated in D2.mdx mice. The expression of endogenous humanin protein was markedly upregulated in the D2.mdx diaphragm compared to the D2.WT diaphragm (Fig. S3).

#### 3.4.4 Serum prednisolone

Serum prednisolone levels were analyzed using mass spectrometry and are presented in Table S1.

#### 3.4.5 Skeletal muscle function

**Fig. 7:**
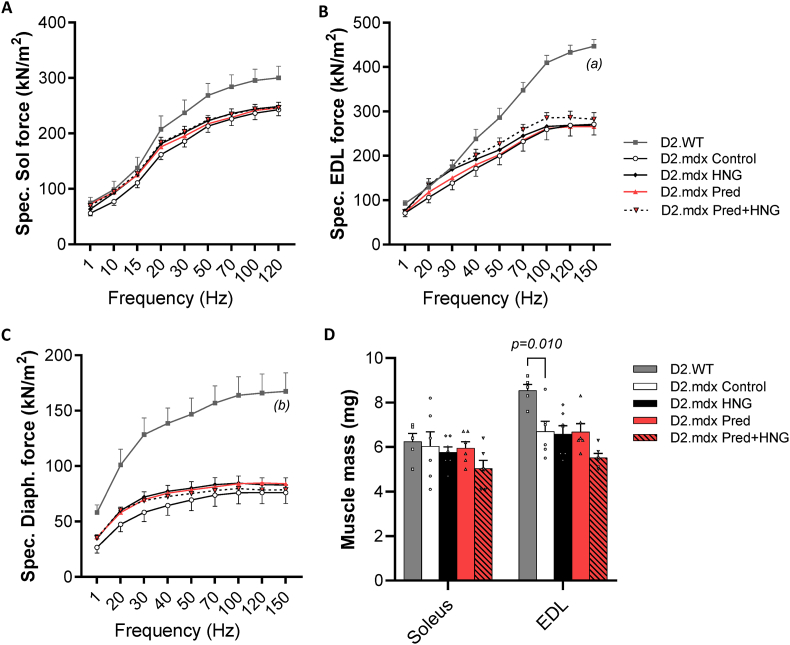
*Ex vivo* force measurements in single muscle fibers isolated from soleus **(A)**, EDL **(B)** and diaphragm **(C)** from D2.WT and treated D2.mdx mice (1 mg/kg BW/day i.p. injections of prednisolone and/or 100 μg/kg BW/day HNG, control = saline). Mean ± SEM, D2.WT: n = 5; D2.mdx treatment groups: n = 6–7. P-values refer to D2.WT vs. D2.mdx control (a) 70 Hz p = 0.023; 100–150 Hz p < 0.01 and (b) 30–150 Hz p < 0.05, mixed-model ANOVA. The muscle masses of soleus and EDL **(D)**, Student's unpaired *t*-test (D2.WT vs. D2.mdx control) and one-way ANOVA between D2.mdx treatment groups (no significant difference).

**Fig. S4:**
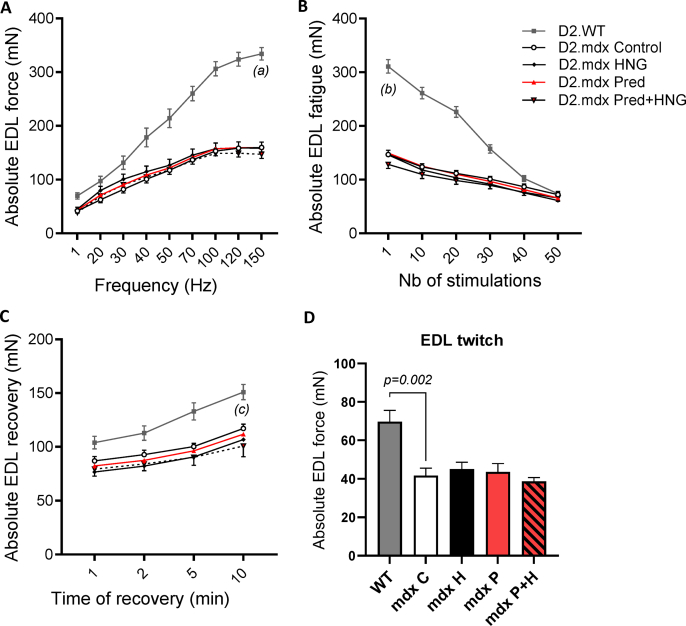
Effects of prednisolone and HNG on skeletal muscle function in EDL muscle. Absolute force (A), fatigue (B), recovery (C) and twitch (D) in D2.WT and treated D2.mdx mice (1 mg/kg BW/day i.p. injections of prednisolone and/or 100 μg/kg BW/day HNG, control = saline). Mean ± SEM, n = 5-7. P-values refer to D2.WT vs. D2.mdx control (a) 1 and 50 Hz: p< 0.05; 70-150 Hz p ≤ 0.001, (b) 1-20 stimulations p < 0.001; 30 stimulations p = 0.003, (c) 5-10 min p < 0.05.

**Fig. S5:**
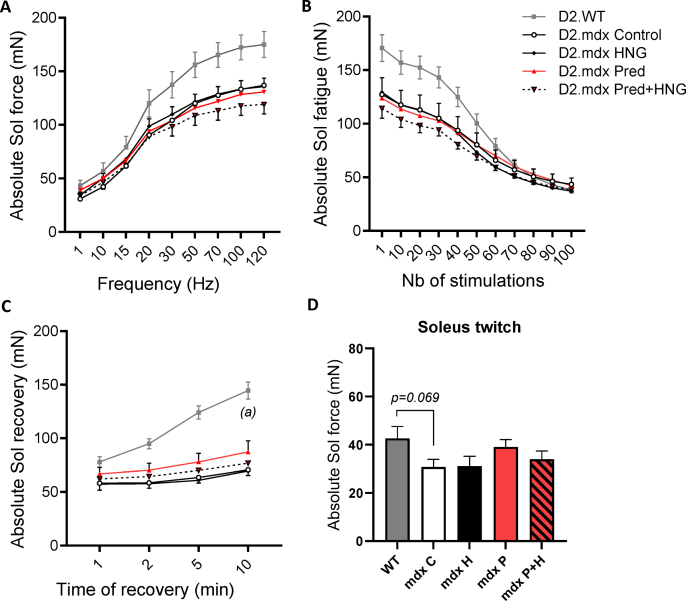
Effects of prednisolone and HNG on skeletal muscle function in soleus muscle. Absolute force (A), fatigue (B), recovery (C) and twitch (D) in D2.WT and treated D2.mdx mice (1 mg/kg BW/day i.p. injections of prednisolone and/or 100 μg/kg BW/day HNG, control = saline). Mean ± SEM, n = 5-7. P-values refer to D2.WT vs. D2.mdx control (a) 2 and 10 min p < 0.01; 5 min p = 0.002.

The effects of prednisolone and HNG on skeletal muscle function in D2.mdx mice were evaluated using *ex vivo* force measurements of the lower limb (EDL and soleus) and respiratory muscles (diaphragm) (Fig. 7, S4-5). A lower dose of prednisolone (1 mg/kg BW/day) was used, as it was previously reported to have positive effects on muscle function in a mdx mouse model [43]. Soleus, EDL and diaphragm muscles from D2.mdx mice were drastically weaker than muscles from D2.WT mice. Neither prednisolone nor HNG or the combined treatment altered the force production. The EDL muscle of all the experimental groups was smaller than that noted in D2.WT mice (Fig. 7D). No difference in muscle size was observed in soleus muscle. Detailed data on absolute force, fatigue, recovery and twitching in the EDL and soleus muscles are presented in Fig. S4 and S5.

## 4 Discussion

The primary objective of this study was to investigate whether systemic treatment with HNG, a synthetic analog of mitochondrial peptide humanin, improves bone health in a GC-treated DMD mouse model. Our findings indicate that HNG treatment improves bone health in GC-treated mdx mice. Notably, the combination of HNG with GCs did not exacerbate skeletal muscle impairment.

We used two strains of dystrophic mice in this study: B10.mdx and D2.mdx. Untreated B10.mdx mice, the most used mouse model for DMD studies, showed no difference in longitudinal tibia growth compared with B10.WT mice. In contrast, D2.mdx mice exhibited growth retardation over the four-week study period, similar to that observed in DMD patients who are generally shorter than the healthy population regardless of GC treatment [[4], [5], [6]].

Bone geometry and mineral content were impaired in D2.mdx compared with WT mice, but not in B10.mdx mice. However, a compromised bone phenotype was previously reported in B10.mdx mice [44]. The D2.mdx model better reflects the severity of human DMD than the B10.mdx model [45], and this model also showed a skeletal phenotype in our study. This finding is similar to what is known in boys with DMD (analyzed with dual-energy X-ray absorptiometry), where long bone deficits occur early, even before GC treatment and loss of ambulation [2,11]. Detailed bone analysis using pQCT has been reported primarily for DMD-patients on GC therapy. The bone phenotype we observed in D2.mdx mice—with reduced cortical thickness and mineral content but preserved cortical BMD—closely mirrors that reported in patients [46]. Although DMD patients also show lower trabecular bone density with a rapid decline after loss of ambulation [46], we found a nonsignificant tendency toward lower trabecular BMD in D2.mdx mice. These data suggest that the D2.mdx mouse strain recapitulates the bone phenotype observed in DMD patients and therefore represents a good model for studying bone health issues associated with DMD.

Owing to the pronounced bone phenotype in the D2.mdx model, we conducted most of our studies using these animals. Interestingly, we detected decreased expression of endogenous humanin in the growth plates of D2.mdx mice. This may be linked to systemic inflammation known to be present in DMD [47] given that a similar decrease in humanin expression has been observed in human growth plates cultured *ex vivo* with the proinflammatory cytokine TNF-alpha or serum from patients with inflammatory bowel disease [48]. Dystrophin deficiency triggers muscle inflammation in D2.mdx mice, and significant increases in the expression of mRNA markers of macrophages, such as *Lgals3* and *Mpeg1*, have been reported [45].

Short stature, worsened by long-term GC treatment, is frequently observed in DMD patients [3,8]. Treatment with HNG has previously been shown to prevent growth retardation caused by chronic inflammatory conditions [48], anticancer drugs [23,49], and GC treatment in healthy mice [24]. In this study, four weeks of treatment with a subcutaneous GC pellet impaired bone growth, and cotreatment with HNG effectively ameliorated this adverse effect during the first week in B10.mdx mice. Interestingly, humanin overexpression has also been shown to protect against GC-induced apoptosis and growth retardation in young mice [24].

Osteoporosis causes painful fractures in DMD patients, which can lead to earlier wheelchair dependence and negatively impact survival [50]. The current treatment strategy for osteoporosis involving bisphosphonates is associated with both acute and long-term adverse effects [19,20]. We demonstrated that in mdx mice, both subcutaneous pellets and intraperitoneally administered doses of GCs induced cortical bone loss. Adverse effects on bone with GC pellets are well known in mdx mice [32,33]; however, analogous data for i.p. injections are limited. We found that cotreatment with 1 mg/kg BW/day HNG preserved cortical bone area and thickness, an effect observed after four weeks of treatment. One mechanism by which GCs induce cortical bone loss involves the release of RANKL by osteocytes, promoting osteoclast formation [51]. Humanin suppresses RANKL-induced osteoclastogenesis *in vitro* [52], which may explain the bone-rescuing effects of humanin reported here.

To exclude any potential side effects of HNG treatment on dystrophic muscle, we conducted several additional analyses. Histological examination revealed a tendency toward larger muscle fibers in D2.mdx mice receiving HNG, either alone or when the combined GC + HNG treatment was compared with the GC treatment alone. Previous reports on the effect of HNG on cell size have focused primarily on its ability to restore cell size under abnormal conditions [49,53], in addition to its positive effects on mitochondrial biogenesis in pancreatic cells [54]. Serum CK activity was, as expected, higher in D2.mdx than in D2.WT, but with no differences between treatment groups. However, our CK data need to be interpreted with caution as cardiac puncture was applied for blood collection, which may increase variability due to sampling-related cardiac muscle damage [55].

Given these findings, we also examined the effect of HNG on skeletal muscle strength *ex vivo*. Soleus and EDL muscles were investigated because they are predominantly slow- and fast-twitch, respectively. The diaphragm was also assessed as the main respiratory muscle. We observed a profound alteration in muscle function in D2.mdx mice, with a specific force lower in D2.mdx mice than in WT mice, irrespective of treatment and muscle type. The effects of long-term treatment with HNG on mobility and strength in dystrophic mice require further investigations through *in vivo* functional tests. Interestingly, chronic HNG treatment reverses cardiac fibrosis and apoptosis in the hearts of aging mice, with no adverse effects reported [56].

This study has several limitations. First, high doses of GCs were used to induce growth retardation and osteoporosis. In our experimental settings, growth retardation occurred only when a slow-release prednisone pellet was used. We hypothesize that the increase in tibia growth seen after two weeks in the PredP group may have resulted from an uneven release of prednisone from the pellet, with a higher release rate initially as reported by others with similar pellets [34,35]. To address this, we modified the protocol to use controlled daily i.p. injections, allowing for dose adjustments on the basis of the body weight of the animals. This route induced changes in cortical bone and body weight but did not significantly inhibit longitudinal tibia growth, making it a less effective model for GC-induced growth retardation. As a result, potential HNG effectiveness could not be properly assessed in this model. The lower dose of prednisolone used, 5 mg/kg BW/day, has been reported to cause shorter tibias in B10.mdx mice [36], but no data are available for the D2.mdx mouse strain. Our data suggest that an earlier start of treatment in even younger animals is needed, as the greater dosage of 10 mg/kg BW/day was also not sufficiently effective.

Secondly, HNG transiently affected bone growth during the first week, while the effects on pQCT-derived bone parameters remained at the experimental endpoint after four weeks. Finally, only a lower dose of HNG, the same dose as that previously shown to rescue growth in mice, was tested in D2.mdx mice [24]. The efficacy and safety of higher doses of HNG need to be further investigated in pronounced DMD models.

In summary, our results suggest that HNG is a promising candidate for improving bone health in DMD during GC treatment. The development of new treatment strategies to support bone health could increase the quality of life for DMD patients, which is essential considering the limitations and side effects of currently available treatments. Given the reported beneficial effects of HNG on cardiac health, it is valuable to expand these findings further. Additional studies are needed to determine the optimal HNG dosage and to examine the effects of long-term treatment on mobility and skeletal muscle strength.

## Funding

TC was funded by Sällskapet Barnavård, Stockholm. LS was funded by grants from the 10.13039/501100004359Swedish Research Council (2024–03265), the 10.13039/501100004047Karolinska Institute, the Swedish Governmental grants under the ALF agreement by RegionStockholm (ALF; RS2021–0855), Stiftelsen Frimurare Barnhuset inStockholm, and from Märta and Gunnar V Philipson Foundation. FvW was funded by a grant from the 10.13039/501100004359Swedish Research Council (2022-01392). TC, FvW, 10.13039/100016851TS, FZ and LS have been supported by a research fund to the Center for Neuromusculoskeletal Restorative Medicine from the Health@InnoHK program launched by the 10.13039/501100003452Innovation and Technology Commission, the Government of the Hong Kong Special Administrative Region of the People's Republic of China. FZ was funded by Åke Wibergs Stiftelse.

## CRediT authorship contribution statement

**Therése Cedervall:** Formal analysis, Investigation, Project administration, Writing – original draft. **Baptiste Jude:** Formal analysis, Investigation, Writing – original draft. **Ferdinand von Walden:** Methodology, Writing – review & editing. **Lilly Velentza:** Investigation. **Johanna T. Lanner:** Writing – review & editing. **Thomas Sejersen:** Conceptualization, Funding acquisition, Supervision, Writing – review & editing. **Farasat Zaman:** Conceptualization, Supervision, Writing – review & editing. **Lars Sävendahl:** Conceptualization, Funding acquisition, Supervision, Writing – review & editing.

## Declaration of competing interest

The authors declare that they have no known competing financial interests or personal relationships that could have appeared to influence the work reported in this paper.

## Data Availability

Data will be made available on request.
