# Supplementary material for: Humanin improves bone health in a glucocorticoid-treated mouse model of Duchenne muscular dystrophy
Source: Biochem Biophys Rep. 2026 Jan 6;45:102421. doi: 10.1016/j.bbrep.2025.102421 (PMC12811480; doi:10.1016/j.bbrep.2025.102421)
Supplement: Multimedia component 1 [file mmc1.docx]

**Supplementary text**

**S1.** Prednisolone was quantified using a Waters UPLC-MS/MS system (Waters, Milford, Mass.)

Separation was done on the ACQUITY I-Class UPLC system using a Waters ACQUITY UPLC BEH C18 1.7µm, 2.1 x 50mm column (Waters, Wexford, Ireland) with a gradient of 0.1% formic acid (FA) in water in mobile phase A and 0.1% FA in acetonitrile in B. The gradient started at 20% B, held for 1 minute followed by a 2-minute linear gradient to 95% B. After a1-minute column wash, the column was equilibrated in 20%B for 1 minute. Mobile phase flow was 0.6mL/min, column temperature 60°C, and a 5uL sample was injected.

MS detection was performed on a Waters XEVO TQ-MS tandem quadrupole mass spectrometer (Waters, Milford, Mass.) in the selected reaction monitoring mode using mass transition 343.2 > 147.0 at 25 collision energy. For 8-deuterated prednisolone as internal standard, transition 351.2 > 151.0 at 28eV was used. Ionization was electrospray with a capillary voltage of 1.5kV and accelerating cone voltage of 25V.

Quantitative analysis was done in TargetLynx software (Waters, Milford, Mass.) using the deuterated prednisolone as internal standard. A 6-point calibration curve spiked in plasma in the relevant concentration range was injected in triplicate bracketing the samples, and low, middle and high-quality control samples were analyzed together with the 16 samples. The coefficient of determination of the calibration curve was 0.998.
