# Supplementary material for: Humanin improves bone health in a glucocorticoid-treated mouse model of Duchenne muscular dystrophy
Source: Biochem Biophys Rep. 2026 Jan 6;45:102421. doi: 10.1016/j.bbrep.2025.102421 (PMC12811480; doi:10.1016/j.bbrep.2025.102421)
Supplement: Multimedia component 2 [file mmc2.docx]

**Supplementary table**

| ***Table S1.*** *Prednisolone concentrations in serum* | | |
| --- | --- | --- |
| 10 mg/kg BW prednisolone  30 minutes after injection | 10 mg/kg BW prednisolone  24 hours after injection | 2.5 mg prednisone s.c. pellet  4 weeks after implantation |
| 1876.4 ± 128.3 ng/mL | 0.4 ± 0.02 ng/mL | 3.9± 0.8 ng/mL |
| *The concentration of prednisolone, analyzed with mass spectrometry, in serum collected from D2.mdx mice injected i.p. with 10 mg/kg BW prednisolone (30 min or 24 hours prior to blood collection), or B10.mdx mice 4 weeks after implantation with a 2.5 mg prednisone s.c. pellet (prednisone is metabolized into its active form prednisolone).* | | |
